# Molecular mechanisms of fission in echinoderms: Transcriptome analysis

**DOI:** 10.1371/journal.pone.0195836

**Published:** 2018-04-12

**Authors:** Igor Yu. Dolmatov, Sergey V. Afanasyev, Alexey V. Boyko

**Affiliations:** 1 A.V. Zhirmunsky Institute of Marine Biology, National Scientific Center of Marine Biology, Far Eastern Branch, Russian Academy of Sciences, Vladivostok, Russia; 2 Far Eastern Federal University, Vladivostok, Russia; 3 Sechenov Institute of Evolutionary Physiology and Biochemistry, Saint Petersburg, Russia; Chang Gung University, TAIWAN

## Abstract

Echinoderms are capable of asexual reproduction by fission. An individual divides into parts due to changes in the strength of connective tissue of the body wall. The structure of connective tissue and the mechanisms of variations in its strength in echinoderms remain poorly studied. An analysis of transcriptomes of individuals during the process of fission provides a new opportunity to understand the mechanisms of connective tissue mutability. In the holothurian *Cladolabes schmeltzii*, we have found a rather complex organization of connective tissue. Transcripts of genes encoding a wide range of structural proteins of extracellular matrix, as well as various proteases and their inhibitors, have been discovered. All these molecules may constitute a part of the mechanism of connective tissue mutability. According to our data, the extracellular matrix of echinoderms is substantially distinguished from that of vertebrates by the lack of elastin, fibronectins, and tenascins. In case of fission, a large number of genes of transcription factors and components of different signaling pathways are expressed. Products of these genes are probably involved in regulation of asexual reproduction, connective tissue mutability, and preparation of tissues for subsequent regeneration. It has been shown that holothurian tensilins are a special group of tissue inhibitors of metalloproteinases, which has formed within the class Holothuroidea and is absent from other echinoderms. Our data can serve a basis for the further study of the mechanisms of extracellular matrix mutability, as well as the mechanisms responsible for asexual reproduction in echinoderms.

## Introduction

Asexual reproduction is the most ancient type of reproduction of organisms that occurs in members of most phyla of modern Metazoa [1–3]. Unlike the studies of embryonic development and regeneration, which are considered in a large number of publications, the ones of mechanisms of asexual reproduction in animals are scarce. Currently, the data on the origin and evolution of this type of reproduction, as well as on genes expression during blastogenesis and transverse division (fission) are available [4–11]. There is only one publication dedicated to the analysis of transcriptome of animals during asexual reproduction [12].

One of the animal phyla that are able to reproduce asexually is Echinodermata. These are ancient, exclusively marine animals, which, along with chordates and hemichordates, form the group Deuterostomia. Asexual reproduction is found in some members of Asteroidea, Ophiuroidea, Echinoidea, and Holothuroidea [13–15]. Sea urchins are capable of asexual reproduction only at the larval stage [14,15]. In adult individuals of sea stars, ophiuroids, and holothurians, it is performed through fission or autotomy. The greatest number of fissiparous species (45) has been recorded in the class Ophiuroidea [13]. The number of species capable of fission among sea stars and holothurians is much lower, 27 and 16, respectively [13,16]. Asexual reproduction in echinoderms is poorly understood as well as in other animals. To date, no studies of the cellular and molecular mechanisms of fission in echinoderms have been conducted. There are a few publications on morphology of dividing individuals and regeneration of fragments after fission [17–21].

The body wall in echinoderms consists almost exclusively of connective tissue [22,23]. Therefore, dividing the body by fission is impossible without transforming the extracellular matrix (ECM). Some information on the structure and properties of echinoderm ECM are available. According to the data of morphological and biochemical studies, connective tissue of echinoderms contains bundles of collagen fibrils, proteoglycans, and fibrillin microfibrils [24–28]. Recently, the genome of the holothurian *Apostichopus japonicus* has been sequenced [29,30]; however, no ECM genes in these species have been analyzed. ECM genes of the sea urchin *Strongylocentrotus purpuratus* are described more in detail. This species has a set of genes for ECM components similar to that of other animals: collagens, proteoglycans, laminins, etc. [31,32]. Moreover, echinoderms possess proteins that modify ECM, such as matrix metalloproteinases (MMP), disintegrin and a metalloproteinase with thrombospondin motifs (ADAMTS), as well as tissue inhibitors of metalloproteinases (TIMP) [33–37].

A noteworthy feature of echinoderm connective tissue is the capability of changing its mechanical properties under the effect of various factors [38,39]. For this reason, it is referred to as mutable collagenous tissue (MCT) [40], or catch connective tissue [41]. Echinoderms use this property for, as an example, maintaining a posture (the catch state) [42,43] and during autotomy [38]. This ability is assumed to also be involved in asexual reproduction [13,44–46]. MCT has been found in all members of extant echinoderm classes [38]. It can form various anatomical structures such as diverse ligaments, as well as connective tissue of the body wall [38,47].

A few hypotheses have been proposed for explaining the mechanisms of MCT changes [36,45,48,49]. All of them are based on the fact that under the influence of some factors there is an increase or decrease in the number of cross-links between collagen fibrils, which make the connective tissue more rigid or soft. The substances that facilitate the transition of MCT from one state to another have been identified and partially characterized [50–53]. Nevertheless, neither the complete amino acid sequence nor the type of these proteins have been determined. The only exception is tensilin, which is believed to stiffen of connective tissue (Keene, Trotter, unpubl., cited by Wilkie [39]). The amino acid sequence of this protein was determined for the holothurian *Cucumaria frondosa* [54]. It was found that tensilin has a high homology to TIMP. The latter finding is in accordance with participation of MMPs in functioning of MCT [39]. Several proteases exhibiting a gelatinase activity have been detected in the compass depressor ligaments of the sea urchin *Paracentrotus lividus* [36]. Blocking of them by a specific inhibitor increased the stiffness of the ligament. In addition, several bioactive peptides capable of changing the stiffness of connective tissue of the body wall in holothurians were found [55–57]. Proteoglycans and a number of other protein complexes are supposed to participate in changing the MCT properties [56]. Nevertheless, it is still unclear which components of connective tissue are responsible for changing the mechanical properties of MCT.

We have recently found that the holothurian *Cladolabes schmeltzii* shows the capability of transverse division [58,59]. The structure of internal organs and their post-fission regeneration in this species have been studied in detail [20,21,60]. The present study considers a comparative analysis of transcriptome of tissues in intact holothurians and individuals in the process of fission. We attempted to identify the transcripts of genes that can theoretically participate in the mechanisms of ECM mutability in case of body division and in the regulation of asexual reproduction in echinoderms.

## Materials and methods

**Fig 1:**
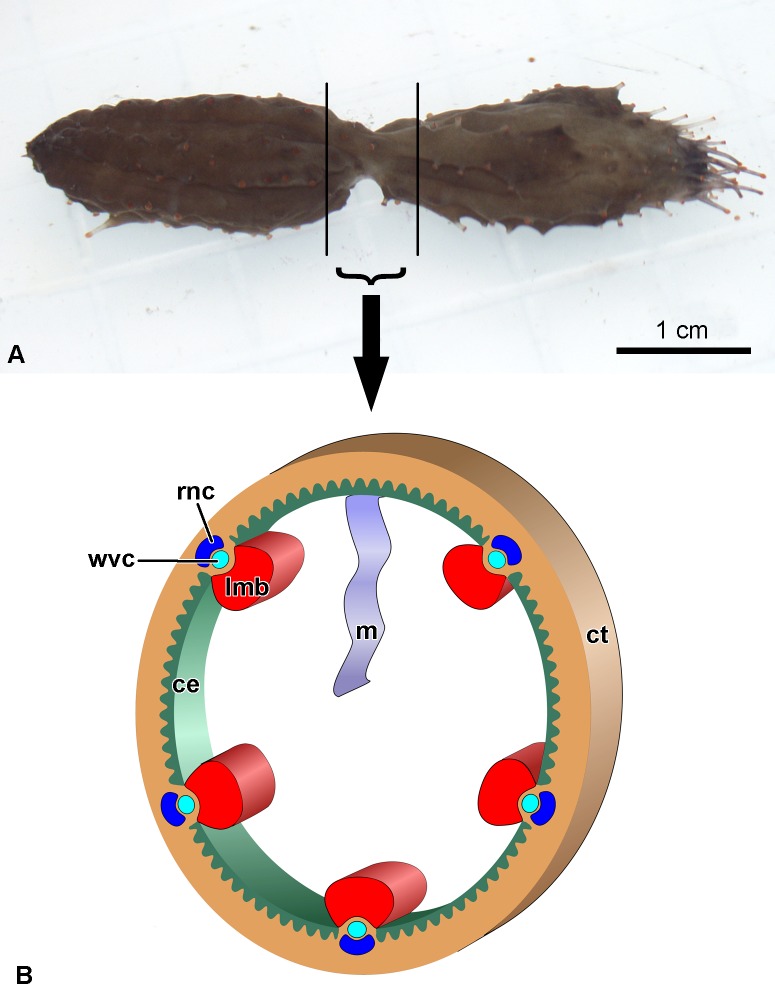
Collection of tissues for experiments. (A) Holothurian *Cladolabes schmeltzii* during fission. Vertical lines bound part of the body which takes for analysis. (B) Structure of the part of the body which takes for analysis. ce, coelomic epithelium; ct, connective tissue of the body wall; lmb, longitudinal muscle band; m, gut mesentery; rnc, radial nerve cord; wvc, radial water-vascular canal.

The study was carried out using adult individuals of the holothurian *Cladolabes schmeltzii* (Holothuroidea, Dendrochirotida). The animals were collected in Nha Trang Bay, South China Sea near the south part of Hon Tre island (12°10ʹ51ʺ, 109°17ʹ35ʺ). *C*. *schmeltzii* are abundant in coastal areas of Vietnam. The species is not endangered or protected. They are invertebrate animals and no specific permissions are required for their collection. In three holothurians that were in the process of division, the area of the body with the constriction formed during fission was taken for the analysis (Fig 1A). Three individuals without signs of division or regeneration were used as the control. The middle part of their bodies was taken for the analysis. In both cases, the body wall with the constituent structures (coelomic epithelium of interradii and ambulacra consisting of the radial nerve cord, water-vascular canal, and longitudinal muscle band), and intestinal mesentery were sampled (Fig 1B). The digestive tube was removed. Samples were placed into an RNAlater and stored at –20°C for 4 weeks.

Samples of intact and dividing animals were treated separately. Tissues of three animals were mixed and homogenized. Total RNA was isolated by extraction in phenol-chloroform with TRIreagent (MRC) by the standard method. Treating with DNAase1 (ThermoScientific) was performed with addition of inhibitor of RNAases RiboLock (ThermoScientific). Synthesis and normalization of cDNA, construction of libraries, library quality control and sequencing were carried out by Evrogen JSC. The amplified dsDNA was prepared using the SMART method. To obtain the most complete transcriptome, the *in vitro* DSN-normalization method was used [61]. Samples of cDNA were prepared for sequencing using NEBNext dsDNA Fragmentase (NEB) and a NEBNext® DNA Library Prep Master Mix Set for Illumina (NEB). The quality control of a sample ready for sequencing (pool of libraries) included determination of concentration using Qubit, qPCR, and testing for Agilent DNA7500 chip. The library pool was sequenced in one run of Illumina HiSeq 2000, 101 cycles, paired-end reads with the use of TruSeq SBS sequencing kit version 3 (Illumina). Data processing was performed using the Casava 1.8.2 software (Illumina). Libraries for sequencing on the 454 GS FLX+ platform were composed using Roche GS Rapid Library Prep Kit.

All reads were filtered and trimmed using the Trimmomatic 0.36 tool with parameters “LEADING:20 TRAILING:20 SLIDINGWINDOW:5:21 AVGQUAL:25 MINLEN:30” [62]. Read pairs, including overrepresented sequences, were removed. Filtered reads were assembled using a SPAdes 3.11.1 tool with 2 iterations of read error correction and three kmer sizes: 49, 33, and 25 [63]. All assembled contigs be used for CDS searching using the TransDecoder 4.1 software with a minimum protein length of 50 amino acids. Code of TransDecoder tool was modified for extracting non-metionin started CDS. Then, all CDS was clustered CD-HIT 4.6 [64,65] with the following parameters “-n 7 -c 0.9 -G 0 -aS 0.8 -A 120” and overassembled using the own Python script ThreadHomoloCAP3. For assembly of sequences with small (> 30 nt) end overlaps, CAP3 were used with “-r 0 -p 95 -o 30 -h 3 -y 10 -t 500 -s 400 -i 32 -j 42” parameters [66]. Then, a SPAdes part “corrector” was used for sequence error correction and removing the erroneous connections that could occur at the last two steps of the assembly. Isoform identification was performed using CD-HIT with the following parameters: “-n 7 -c 0.9 -G 0 -aS 0.5 -A 150”.

The assembled sequences have been deposited in the NCBI Transcriptome Shotgun Assembly (TSA) database (GFWR00000000) and NCBI Sequence Read Archive (SRA) (SRR6023958, divided holothurians; SRR6023959 and SRR6425862, intact holothurians). The assembled contigs were used as the input for the BLASTX homology search [67] against the NCBI non-redundant protein database with the e-value threshold of 1e-5. The best hit was determined by bitscore value. Alignment and calculation of the number of mapped Illumina paired-end reads were performed using the Trinity 2.4.0 scripts [68], Bowtie 2.2.9 [69], and RSEM 1.3.0 [70]. The following parameters were added to the default ones: “-L 25 -N 1 –minins 50 –maxins 600”. A BLAST search against SwissProt database was used for obtaining the GO terms. The GO Enrichment analysis was conducted using GOAtools 0.5.9 [71].

For finding the unique contigs for fission and norm, we used the number of paired-end reads per contig [72]. If this number is enough for a 10-fold coverage of contig, the latter is considered as present in the sample being analyzed. This threshold was introduced due to the impossibility to assess expression in sample after DSN-normalization and determined empirically.

The domain structure of the supposed proteins was determined using the SMART (http://smart.embl-heidelberg.de/) and NCBI’s conserved domain search tool. The study of potential TIMP sequences was performed with HMMER 3.1 [73] against the Pfam domain database. TIMP sequences of echinoderms and *Crassostrea gigas* (outgroup) [37], sea urchin *S*. *purpuratus*, sea star *Patiria miniata*, and holothurian *Parastichopus parvimensis* [74,75], three tensilin sequences from NCBI (PIK52999, PIK53591, and AQR59058), and TIMP sequences of *C*. *schmeltzii* were used for the analysis. The sequences were filtered by the minimum alignment length of 130 amino acids and verified by the NCBI NR protein database. Alignment was created using COBALT with standard settings [76]. All sequences distorting the alignment were removed. Gblocks 0.91 with minimum settings was used for removing bad blocks from alignment [77]. Then all the amino acids of the sequences in alignment were replaced by the corresponding triplets from the original nucleotide sequences. For choosing the optimal settings of tree computing, PartitionFinder 2.1 was used [78]. Tree computing was performed by means of the PhyML 3.3 tool [79].

The results were obtained using the equipment of Shared Resource Center “Far Eastern Computing Resource” of Institute of Automation and Control Processes FEB RAS.

## Results and discussion

### Transcriptome sequencing and annotation

A total of 37.6 million Illumina paired-end reads and 237 thousand 454 GS FLX+ single reads were obtained as a result of sequencing of the sample from intact (control) holothurians and 53% raw reads remained after filtration. When sequencing the sample of dividing animals, we obtained a total of 61.8 million Illumina paired-end reads and 32% raw reads remained after filtration. The assembly includes 50959 contigs with a mean length of CDS 516 nt (S1 Fig). Of all contigs, 37% have significant BLAST hits; 3042 contigs have only unnamed best hits. The results of the BLAST analysis are listed in S1 Table. A total of 3301 unique contigs were found in tissues of intact animals; in dividing holothurians, 13 322. Of these, there are hits in the NCBI NR protein database: 2670 and 5324 contigs, respectively.

### GO annotation

**Fig 2:**
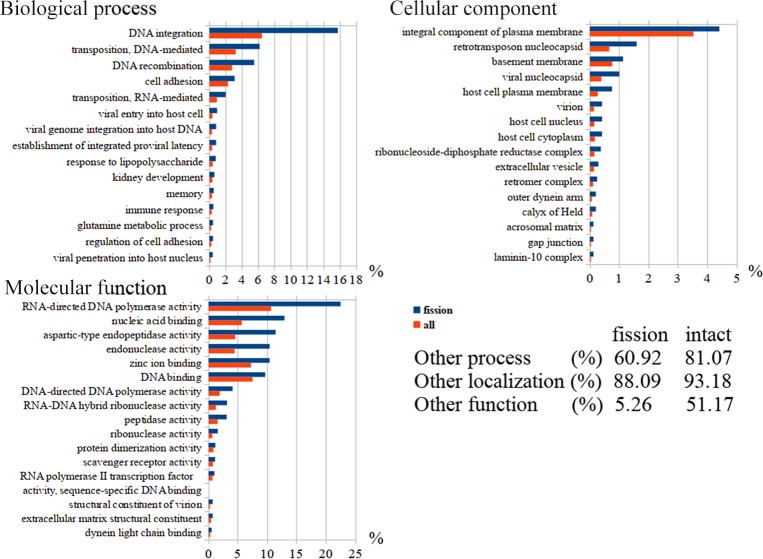
GO enrichment analysis of genes in *Cladolabes schmeltzii*.

An analysis of gene ontology showed that the physiological condition of holothurians quite markedly changes in case of fission (Fig 2). Such biological processes as metabolism, cell adhesion, and immunity are activated in the animals. In addition, dividing holothurians increase the number of genes with the GO term “memory”. This probably indicates the involvement of the nervous system in the asexual reproduction process. This assumption is confirmed by an increase in genes with the GO term “calyx of Held” (cellular component) in dividing animals.

Among the molecular functions, a significant increase in peptidase activity during fission should be noted. Genes having a similar function are associated with such GO terms as “aspartic-type peptidase activity”, “zinc ion binding”, and “peptidase activity”. The intensification of peptidase activity during fission and the involvement of aspartyl peptidases in this agree with the previously obtained data on the holothurian *A*. *japonicus*. In this species, cathepsin D (aspartyl peptidase) takes an active part in degradation of extracellular matrix of the body wall [80].

Among cellular components, we can emphasize the genes grouped under the term “integral component of plasma membrane”, which hints at the variations in receptor complexes of cells at the site of fission. The active transformation of organs and extracellular matrix is indicated by such terms as “basement membrane”, “gap junction”, “laminin-10 complex”, and “extracellular vesicle”. In general, GO annotation shows that many physiological and structural changes occur in the process of asexual reproduction. In holothurians, metabolism, immune and nervous systems are activated.

It is an interesting fact that transcripts of genes, associated with the functioning of viruses and retrotransposons, predominate in tissues of dividing individuals of *C*. *schmeltzii*. For example, in the Biological process group, they account for about 33%. As is known currently, retrotransposons may participate in regulation of various functions of organism [81,82]. Their increased expression is observed under a stress, in development and regeneration [83–85]. Our data show that retrotransposons are activated also in asexual reproduction and, probably, take some part in regulation of fission in echinoderms.

### Components of extracellular matrix

In *C*. *schmeltzii*, transcripts of the genes of many ECM components such as collagens, proteoglycans, and glycoproteins, which are characteristic of most multicellular animals [86], have been identified both in intact and dividing animals. At the same time, the differences in connective tissue of echinoderms and vertebrates were revealed. In particular, one of the main components of the vertebrate ECM is elastin, the fibers of which are formed by polymerization of tropoelastin [31]. To our surprise, we did not find the products of the *tropoelastin* in the transcriptome of *C*. *schmeltzii*. An analysis of the NCBI databases has shown an absence of sequences close to *tropoelastin* in echinoderms. An additional search in the genomes of the sea urchin *S*. *purpuratus* [74] and the holothurian *A*. *japonicus* [30] has also brought no results. It may indicate the absence of this gene in echinoderms. Another distinguishing feature of echinoderm ECM is the lack of tenascins and fibronectins [32,87]. These proteins play an important role in the structural integrity of connective tissues in vertebrates [88,89]. Our analysis of the domain structure of echinoderms “fibronectins” has shown that they lack the domains FN1 and FN2 typical of chordates. Their molecule consists of only a few FN3 domains.

The transcripts that are blasted as tenascin-like proteins were found in *C*. *schmeltzii* (S1 Table). These contigs encode domains FBG and TILa that are characteristic of tenascins. Recently, it was shown that the holothurian *A*. *japonicus* has tenascin-like proteins, which contain the EGF and FBG domains [90]. Nevertheless, according to Hynes [91], all these domains are ancient in origin and occur in many of animals; however, the combination typical of tenascins is observed only in chordates. Thus, the absence of proteins such as tropoelastin, fibronectins, and tenascins indicates significant differences in the organization of connective tissue in echinoderms and chordates.

### Collagens

The total number of types of collagens found in *C*. *schmeltzii* is difficult to estimate, since the identified contigs often show similarities with genes of several types of close collagens. Nevertheless, members of almost all major groups of collagens were revealed in this species: fibril-forming collagens, fibril-associated collagens with interrupted triple helices (FACIT), network-forming collagens, and multiplexins. Like other echinoderms [30,32], *C*. *schmeltzii* has genes of fibrillar collagens of the types I/II/III and V/XI. Though the relatively small fragments were found in the transcriptomes of *C*. *schmeltzii*, it can be concluded that collagens of this species have a typical structure. Molecules include triple helical domains (Gly-X-Y) and COLFI domain is located at the C-terminus, as in other animals [92].

The FACIT group in *C*. *schmeltzii* is represented by *collagen IX*. In vertebrates, this type of collagen is a component of cartilage, where it is located on the surface of collagen fibrils [93]. Collagen IX molecules are thought to form the macromolecular bridges between the fibrils and other matrix components in cartilage, which is important for the cohesive and compressive properties of cartilage [93]. In echinoderms, collagen IX may have a similar role in some of connective-tissue structures such as ligaments. The ability to form transverse bridges suggests that it can be involved in changes of the mechanical properties of MCT.

The group of network-forming collagens is represented by collagen IV. This type of collagen is a component of basal membranes [31,92,94]. Collagens XV and XVIII form the group of multiplexins. Echinoderms apparently have only one gene, *collagen XV/XVIII* [30,32]. Fragments of transcripts of *collagen XV/XVIII* were detected in the transcriptome of *C*. *schmeltzii*. Collagens XV and XVIII are characterized by the highly interrupted collagenous domain and a large number of sites of binding with chondroitin sulfate and heparin sulfate glycosaminoglycans [95]. As a result, their molecules have a complex ‘knot/figure-of-eight/pretzel’ configuration. This structure may serve as a biological ‘spring’ to stabilize and enhance resilience to compressive and expansive forces [95]. In echinoderms, collagen XV/XVIII may also be a component of MCT and play a certain role in providing elasticity of ligaments.

There are qualitative differences in the expression of collagen genes between intact and dividing *C*. *schmeltzii* (Table 1, S1 Table). Products of *collagen IV* and *collagen XV/XVIII* are found only in dividing holothurians. The expression of these genes is probably related to the rearrangement and restoration of the connective-tissue structures at the site of fission: basal membranes of the epithelia (*collagen IV*) and ECM of body wall (*collagen XV/XVIII*).

**Table 1:** Genes of structural components of ECM expressing in intact and dividing individuals of *C*. *schmeltzii*.

Gene	intact	fission
*aggrecan*	+	+
*agrin*	+	-
*bamacan*	+	-
*cartilage oligomeric matrix protein*	+	+
*chondroitin sulfate synthase*	+	+
*chondroitinase*	+	+
*collagen V/XI*	+	+
*collagen I/II/III*	+	+
*collagen IV*	-	+
*collagen IX*	+	-
*collagen XV/XVIII*	-	+
*dystroglycan*	+	-
*fibrillin*	+	+
*fibulin*	-	+
*glypican*	+	+
*heparanase*	+	+
*hyaluronidase*	+	+
*laminins*	-	+
*nidogen*	-	+
*perlecan*	-	+
*syndecan*	+	**-**
*thrombospondin 1*	+	+
*thrombospondin 4*	+	+

### Proteoglycans and glycoproteins

Proteoglycans and glycoproteins are multifunctional components of connective tissue, which mediate adhesion, proliferation, differentiation, and migration of various cells [96–98]. The difference between them is relatively arbitrary. In this article, we differentiate them in accordance with Hynes and Naba [31].

The transcripts of genes of a number of proteoglycans found in *C*. *schmeltzii*, were typical for echinoderms [30,32]. These are *syndecan*, *glypican*, *betaglycan*, *bamacan (structural maintenance of chromosomes 3)*, and *perlecan (basement membrane-specific heparan sulfate proteoglycan core protein-like)*. Moreover, in holothurians, we detected products of *aggrecan*, which had not been found in *S*. *purpuratus* [32]. At the same time, transcriptome of *C*. *schmeltzii* lack the products of *Secreted modular calcium-binding protein 1*, which are present in *S*. *purpuratus*. These genes are probably not expressed in tissues of holothurians taken for the analysis.

Of the above-listed proteoglycan genes, *perlecan* is worth special mentioning, as it is expressed only in dividing individuals (Table 1, S1 Table). Its activity, along with expression of *collagen IV* and other genes encoding the proteins of basal membranes (see below), indicates a reorganization of epithelia during fission.

Transcripts of genes of various glycoproteins–*laminins*, *nidogens*, *fibrillins*, *fibulins*, *agrin*, *dystroglycan*, and *thrombospondins*–were detected in *C*. *schmeltzii*. In particular, transcripts of *laminins*, as well as *nidogen/entactin* were found (S1 Table). The proteins encoded by these genes, along with collagens and perlecan, are included in the basic set of “basement membrane ECM toolkit”, typical of all Bilateria [32]. All these genes are expressed in dividing individuals of *C*. *schmeltzii*, which indicates a large-scale rearrangement in coelomic epithelium of the body wall at fission (Table 1, S1 Table).

The transcriptomes of *C*. *schmeltzii* contain products of *fibrillin* genes. Fibrillins are the most important component of connective tissue [99]. In vertebrates, they are secreted in the ECM and form microfibrils, which are likely to form a basis for deposition of elastin [100]. The presence of fibrillin microfibrils, apparently, provides the organ with the ability to strain energy storage and elastic recoil [101,102]. In echinoderms, fibrillins form a network consisting of microfibrils with a diameter of 10–14 nm, which surrounds and penetrates bundles of collagen fibrils [25]. It is assumed that fibrillin microfibrils can participate in constriction of ligaments in sea urchins [103].

Recently, it has been shown that fibrillin microfibrils play an important role in functioning of ECM [101]. They participate in distribution, accumulation, and modulation of the signals of transforming growth factor-beta (TGF-β) and bone morphogenetic protein (BMP), which regulate various aspects of cell activity, including ECM formation and remodeling [104]. In addition, fibrillins can bind to integrin receptors and a number of other molecules, and, as a result, the signals about changes in the extracellular microenvironment are transmitted to cell. In fact, fibrillin microfibrils form niches accumulating various factors [105]. The importance of these niches for the normal functioning of ECM is confirmed by data on mutations of *fibrillins*, which cause a disturbance in the structure of microfibrils. For example, Stiff Skin Syndrome is caused by a mutation in one of the fibrillin domains, which mediates the relation with integrins [106].

In the holoturian *C*. *schmeltzii*, fibrillins are expressed in both intact and dividing individuals (Table 1, S1 Table). It is obvious that in the absence of elastin, fibrilin becomes particularly important in the formation and renewal of ECM in echinoderms. In addition, this protein, apparently the same as in vertebrates, participates in the TGF-β signaling pathway and, through it, can have an influence on changes in ECM properties.

Fragments of transcripts of the *fibulin* genes were found only in dividing individuals of *C*. *schmeltzii* (Table 1). Fibulins are able to bind to many components of ECM, in particular fibrillin, and play an important role in stabilizing the supramolecular complexes of connective tissue [107]. In this respect, they are of certain interest as possible participants in the mechanisms changing the mechanical properties of MCT. It is shown that fibulin 1 accelerates ADAMTS-mediated proteolysis of aggrecan and, thus, participates in tissue renewal [108]. In mammals, fibulins together with fibrillins can bind to latent-transforming growth factor beta-binding proteins (LTBPs) and activate the TGF-β signaling pathway [109–111].

Both in dividing and in intact individuals of *C*. *schmeltzii* fragments of transcripts of the three *thrombospondin* genes were found: *THBS 1*, *THBS 4*, *THBS 5 (cartilage oligomeric matrix protein*, *СОМР)* (Table 1, S1 Table). Thrombospondins (TSPs) play a certain role in the organization of ECM, since they are able to serve as molecular bridges between various components of connective tissue [112]. It was shown that they interact with MMP, fibrillar collagen, TGF-β. TSP-1 and TSP-2 may inhibit activity of MMP2 [113] and regulate its level in the extracellular matrix [114,115]. COMP is capable of binding to collagens II and IX with high selectivity [116,117]. According to Halász et al. [118], СОМР is not related with mature collagen fibrils, and its role is limited only to acceleration of fibrillogenesis. Nevertheless, Geng et al. [117] suggest that COMP can participate in the formation of cross-links between collagen fibrils. Thus, TSPs interacts with molecules that can be involved in mechanisms changing MCT stiffness in echinoderms.

### Polysaccharides

The composition of connective tissue of animals includes polysaccharides such as hyaluronic acid and heparan sulfate. It has been shown that the highly sulfated chondroitin sulfates (CS-GAGs) in MCT structures of crinoids, echinoids, and holothuroids are located along collagen fibrils [119–122]. In this regard, enzymes that synthesize and degrade polysaccharides play a major role in the modification of ECM. The products of the *hyaluronidase*, *chondroitin sulfate synthase*, *chondroitinase (N-acetylgalactosamine-6-sulfatase)*, and *heparanase* genes were found in both dividing and intact individuals of *C*. *schmeltzii* (Table 1, S1 Table). In this regard, it can be assumed that modification of polysaccharides plays a certain role in changing the properties of connective tissue in echinoderms. Nevertheless, it has been shown that enzymes that disintegrate hyaluronic acid and sulphated glycosaminoglycans (hyaluronidase and chondroitinase, respectively) do not affect the mechanical properties of MCT in sea urchins [56].

### Proteins modifying ECM

#### Collagen formation

The synthesis of ECM and changes in its properties depend primarily on enzymes that are responsible for assemblage of various types of fibrils forming the basis of connective tissue. A search for the genes of enzymes involved in formation of collagen fibrils–transglutaminase-2 [123] and lysyl oxidase (Lox) [124,125] was carried out in echinoderms. An analysis of the NCBI databases has shown that echinoderms probably possess one *transglutaminase* and one *Lox* gene. No products of the *transglutaminase* were found in *C*. *schmeltzii*. *Lox* transcripts were present in the transcriptome of *C*. *schmeltzii* and are found in both dividing and intact individuals (Table 2, S1 Table). Judging by the present complete transcript, Lox in holothurians has a typical structure [124] and is synthesized as a pre-protein containing the propeptide domain at the N-terminus, behind which the catalytic domain is located.

**Table 2:** Genes of proteases and their activators and inhibitors expressing in intact and dividing individuals of *C*. *schmeltzii*.

Gene	intact	fission	Gene	intact	fission
*72kDa type IV collagenase*	+	-	*serine protease 2*	-	+
*ADAMTS5*	+	+	*serine protease 3*	-	+
*ADAMTS7*	-	+	*serine protease 4*	-	+
*ADAMTS9*	-	+	*serine protease 5*	+	+
*ADAMTS13*	+	-	*serine protease 6*	+	+
*ADAMTS14*	+	-	*serine protease 7*	+	+
*ADAMTS18*	+	+	*serine protease 8*	+	+
*cathepsin D*	-	+	*tensilin 1*	+	+
*cathepsin L*	+	+	*TIMP1*	+	+
*collagenase 3*	+	+	*TIMP4*	+	+
*collagenase 3–1*	+	-	*TIMP5*	+	+
*furin*	+	+	*TIMP6*	+	+
*lysyl oxidase*	+	+	*TIMP7*	+	+
*MMP14*	+	+	*TIMP8*	+	+
*MMP16-1*	+	+	*TIMP9*	+	-
*MMP24*	+	-	*TIMP10*	-	+
*MMP24-1*	+	-	*TIMP11*	+	+
*serine protease 1*	-	+	*α-2-macroglobulin*	+	+

The mechanical properties of structures containing fibrillar collagens largely depend on cross-links, which are formed due to the activity of transglutaminase-2 and Lox. For example, the stiffness of connective tissue, observed in case of various carcinomas and other diseases, is probably a result of increased activity of *Lox* [126–128]. On the other hand, a reduction in Lox expression decreases the stiffness of ECM and prevents fibrosis [129]. It is probable that different types of connective tissue with various mechanical properties are formed in echinoderms due to regulation of the activity of *transglutaminase* and *Lox*. For example, the body wall in many of holothurians consists of several layers of connective tissue, every of which has a different density and amount of collagen [22,23,130,131]. It seems likely that formation of ligaments in crinoids and echinoids, containing MCT, also depends on Lox and transglutaminase activity.

#### Proteases

A broad variety of proteases capable of degrading ECM proteins were found in the transcriptome of *C*. *schmeltzii*. These are serine, cysteine, aspartyl, and metal peptidases. It is known that serine proteases can destroy connective tissue proteins [132]. Products of 8 genes of serine proteases were found in *C*. *schmeltzii* (S1 Table and S1 File). Serine proteases of *C*. *schmeltzii* differ from one another by presence or absence of the domains Peptidase inhibitor I9 (at N-terminus) and CUB (C-terminus). Transcripts of four genes are found only in dividing individuals (Table 2, S1 Table), which may indicate their involvement in the processes of destruction of the body wall during fission. This assumption is supported by data on the holothurian *A*. *japonicus*. It was shown that serine protease PCSK9 effectively destroys a holothurian collagen even at 4°C [133].

Among cysteine proteases in *C*. *schmeltzii*, we found products of *cathepsin B* and *cathepsin L* genes (Table 2, S1 Table). Cathepsins B and L are lysosomal proteases localized in cells. However, they can be secreted into ECM and digest connective tissue proteins [132,134]. In *A*. *japonicus*, cathepsin L-like protein is detected in epidermis and cells located in the external layer of dermis [135]. It is assumed to participate in the processes of autolysis of connective tissue of the body wall of holothurians. In *C*. *schmeltzii*, the number of aligned paired-end reads per contig for the *cathepsin B* gene was small in both samples, and this gene was removed from the analysis. Contigs of *cathepsin L* are revealed in both intact and dividing individuals. In this connection, it is a potential candidate for participation in the mechanisms of transformation of connective tissue during fission.

Products of *cathepsin D* were detected in the transcriptome of *C*. *schmeltzii*. The same as cathepsin L, aspartyl protease cathepsin D is also a lysosomal enzyme. It has been shown recently that cathepsin D in *A*. *japonicus* participates in autolysis of body wall, muscles, and gut [80]. In *C*. *schmeltzii*, products of this gene are found only in dividing individuals (Table 2, S1 Table). Thus, cathepsin D is likely to be involved in degradation of connective tissue at the site of fission in case of asexual reproduction in echinoderms.

**Fig 3:**
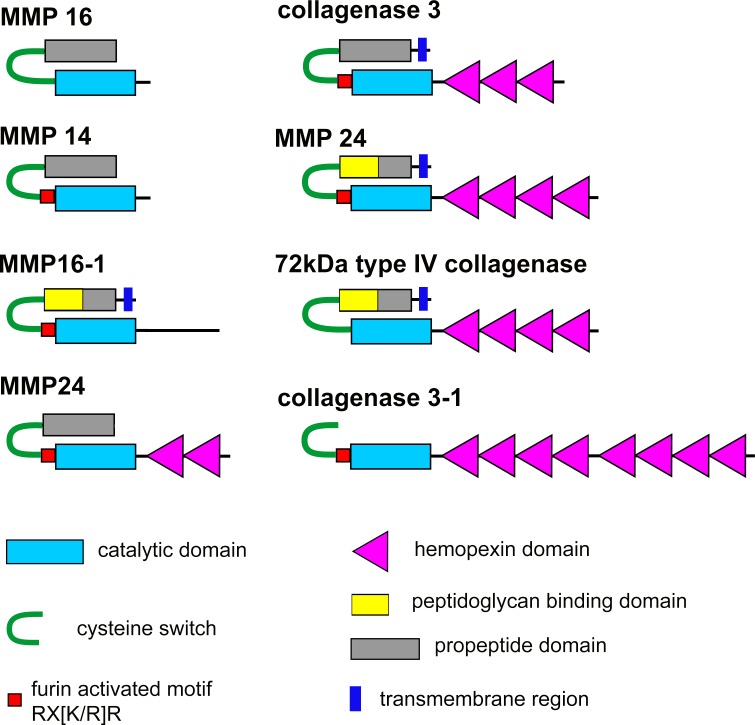
Scheme of structure of the matrix metalloproteinases of *C*. *schmeltzii*.

In works dedicated to the study of MCT of echinoderms, much attention is paid to MMPs [34–36]. They are supposed to participate in mechanisms of MCT mutability. In this regard, we also paid special attention to these proteases. Products of eight MMP genes were identified in the transcriptome of *C*. *schmeltzii* (S1 Table and S2 File). The names of proteinase genes in *C*. *schmeltzii* are quite provisional, as no classification of MMP in holothurians has been developed, and most of these MMPs are identified by a BLAST analysis as MMP16 of the holothurian *A*. *japonicus*. In this regard, MMPs of *C*. *schmeltzii* were denoted analogously to the most closely related MMPs of the sea urchin *S*. *purpuratus*. Seven contigs are full transcripts. They encode proteins consisting of the propeptide domain and catalytic domain (Fig 3). Of them, five proteases contain chemopexin domains at the C-terminus. All the found contigs apparently encode inactive forms of proteases, zymogenes, since the conserved sequence PRCGXXD (cysteine switch) is detected in the predicted amino acid sequences. Cysteine, contained in the cysteine switch, interacts with zinc of the catalytic domain and inactivates its proteolytic activity [136]. The contig encoding collagenase 3–1 probably lacks the N-terminus fragment (Fig 3). Moreover, in six proteinases of *C*. *schmeltzii*, furin activated motif RX[K/R]R is revealed at the C-terminus of the propeptide domain. The presence of this motif indicates that these proteases may be activated by furin [137].

The SMART reveals the transmembrane domain (TD) in four MMPs of *C*. *schmeltzii* (Fig 3). It means that these MMPs may be analogous to the membrane-type matrix metalloproteinases (MT-MMPs) of vertebrates [137]. The presence of MT-MMP-like proteases is suggested for sea urchins also [33]. By means of TD, this protease type can attach to the cytoplasmic membrane and be located on the cell surface. TD plays an important role in functioning of TM-MMPs in vertebrates [137]. Nevertheless, MMPs with TD of *C*. *schmeltzii* differ in structure from MT-MMPs. First, TD in MMPs of *C*. *schmeltzii* is located at the N-terminus of molecule, but not at the С-terminus as in MT-MMPs of vertebrates. In sea urchins, TD is also localized at the N-terminus, for example MMP16 (NM_001033648.1) and 72 kDa type IV collagenase (XM_775263.4). Second, the catalytic domain MT-MMPs contains an insertion of approximately eight amino acids, the so-called MT-loop, which plays an important role in functioning of these proteases [138,139]. In MMPs of *C*. *schmeltzii*, irrespective of presence of TD in them, this insertion is absent.

In *C*. *schmeltzii*, four MMPs (Table 2, S1 Table) are detected only in intact individuals, and they probably do not participate in the ECM transformation at fission. Products of the three protease genes are found in both intact and dividing animals. For this reason, these MMPs can be considered as candidates for participation in mechanisms changing the properties of MCT.

Besides MMPs, the contigs that are fragments of products of *a disintegrin and metalloproteases* (*ADAMs*) and *ADAMTSs* genes were also detected in *C*. *schmeltzii* (S1 Table). Unlike ADAMs, ADAMTSs are mainly responsible for degradation of ECM components, particularly proteoglycans [132]. Products of 7 *ADAMTS* were detected in *C*. *schmeltzii*. Transcripts of *ADAMTS7* and ADAMTS9 are found only in dividing individuals (Table 2, S1 Table). Contigs of another two genes, ADAMTS5 and ADAMTS18, were present in both intact animals and dividing ones. Thus, ADAMTS may be involved in degradation of connective tissue during asexual reproduction.

#### Regulation of protease activity

The activity of MMPs can be controlled in various ways: for example, through regulation of gene transcription, activation of proenzyme by removing the propeptide domain, and interaction with inhibitors [140]. In vertebrates, many of the *MMP* genes are sensitive to a wide spectrum of molecules [140–143]. Promoters of *MMP* genes in mammals contain a TATA box and an activator protein-1 (AP-1) site, with which the transcription factors of the Fos and Jun families can interact [143]. In addition, the promoters contain transcription-binding sites, which jointly regulate gene expression. Data on the structure and regulation of expression of protease genes in echinoderms are not available, and, thus, we can only suppose the possible ways of their activation. In the future, the further analysis of echinoderms’ genomes will provide an opportunity to establish the structure of *MMPs* genes and their promoters and judge more objectively about regulation of *MMPs* expression in these animals. It should be mentioned that transcripts of one of the genes that regulate the expression of *MMPs* in mammals, transcription factor NF-κB, in *C*. *schmeltzii* is found only in dividing individuals (see below).

MMPs are activated through removing the propeptide [140]. This may involve a variety of serine proteinases, such as furin and plasmin, as well as other MMPs. In the transcriptome of *C*. *schmeltzii*, there are products of the *furin* and *plasminogen* genes (S1 Table). Furin can activate metalloproteinases containing furin activated motif (Fig 3). In *C*. *schmeltzii*, products of this gene occur both in intact and in dividing individuals (Table 2, S1 Table). Transcripts of *plasminogen* are not detected at fission.

The inhibitors of metalloproteinases are α-2-macroglobulin, reversion-inducing cysteine-rich protein with Kazal motifs (RECK), and TIMPs [144]. Products of *α-2-macroglobulin* and *TIMPs*, were found in *C*. *schmeltzii* (S1 Table). Transcripts of *α-2-macroglobulin* are found both in intact holothurians and during fission (Table 2, S1 Table).

TIMPs are one of the most important inhibitors of MMPs [145]. The number of *TIMPs* genes in echinoderms varies within a broad range and may reach 45 in some of species [37]. Products of 13 *TIMP-like* genes were identified in *C*. *schmeltzii* (S3 File). Thus, in terms of the number of TIMP genes, this species is inferior to the other studied holothurian species [37], despite it is capable of fission. Predicted amino acid sequences of the TIMPs of *C*. *schmeltzii* are typical of echinoderms [37]. By using the data of Clouse et al. [37], as well as TIMPs sequences of *C*. *schmeltzii* and *A*. *japonicus*, we have built a TIMP tree of echinoderms (S2 Fig). The obtained tree shows that TIMPs of *C*. *schmeltzii* are clustered in different groups and, apparently, represent different types of TIMPs

To date, the mechanisms of interaction of TIMP with MMP have been studied only in mammals. It has been shown that TIMP is characterized by three structural features that ensure the function of these proteins, i.e. binding to MMP molecules [145]. First, it is the presence of the C-X-C motif at the N-terminus, in which one amino acid residue is located between the first and second cysteines. The function of this motif is to interact with a special region of MMP, which plays a major role in determining the specificity of protease. Second, the TIMP molecule contains 12 conservatively arranged cysteine residues, which form the tertiary protein structure due to the formation of disulfide bonds. Third, it is the presence of regions of binding with MMPs, the so-called metzincin-binding and chemopexin-binding interfaces. Variations in the amino acid sequence may lead to a disturbance of the TIMP function. In particular, the inclusion of an additional amino acid between the first and third cysteines results in a disturbance of TIMP’s ability to bind with MMP [145].

Most of the 144 TIMPs of echinoderms, analyzed by us, have 11–12 conservative cysteines (136 sequences) and the standard C-X-C motif (121 sequences) (S3 File). Metzincin-binding interface is recorded only from 46 proteins. The chemopexin-binding interface has been found in none of the TIMPs. It is worth mentioning that most TIMPs with metzincin-binding interface have 11–12 conservative cysteines and the C-X-C motif, i.e. they bear the strongest resemblance with TIMPs of vertebrates. The only exception is 5 proteins: TIMP2c of *S*. *purpuratus* (WHL22.304756.0), TIMPs of *Synapta maculata* (8.m.2049.240066) and *Abyssocucumis abissorum* (43.aa.8353.697), tensilin of *C*. *frondosa* and tensilin2 of *C*. *schmeltzii* (see below about tensilins). Thus, out of the 144 analyzed sequences, as many as 41 TIMPs of echinoderms can be considered as closest to TIMPs of vertebrates in their structure. In *C*. *schmeltzii*, only three TIMP-like proteins–Cs-TIMP6, Cs-TIMP7, and Cs-TIMP8 –have three features specific to mammalian TIMPs (S3 File and S2 Fig). The rest of the proteins have an additional amino acid residue between initial cysteines and/or a missing metzincin-binding interface.

Thus, most echinoderm TIMPs quite significantly differs in structure from mammal TIMPs. These differences are supposed to be related to “co-evolution” with MMPs. In echinoderms, *MMPs* underwent substantial duplication and divergence after the separation of Ambulacraria and Vertebrata [33]. Accordingly, there was also a divergence and TIMPs, which “adapted” to the corresponding metalloproteinases. It is possible that the mechanism of interaction of TIMPs with MMPs also changed, which influenced the structure of these proteins. The increase in the number of genes of MMPs and TIMPs occurred, apparently, due to the increased role of connective tissue in the vital activity of echinoderms. Clouse et al. [37] believe that a large number of TIMPs genes in holothurians are associated with the involvement of these proteins in the control of fission and autotomy. However, autotomy is widely represented not only in holothurians, but also in crinoids, asteroids, and ophiuroids. The number of fissiparous species of sea stars and brittle stars is significantly larger than that of holothurians [13,16]. In this regard, additional studies of the functions of TIMPs in echinoderms and the participation of these proteins in the ECM transformation are required. Moreover, it cannot be ruled out that TIMPs in echinoderms, the same as in vertebrates [144,146–148], can be involved in a wide range of biological functions. This could also have an effect on the number and structure of TIMPs in echinoderms.

It is evident that TIMPs play a certain role in asexual reproduction in *C*. *schmeltzii*. Contigs of *Cs-TIMP10* are recorded only from dividing individuals (Table 2, S1 Table). Furthermore, most of *TIMPs* genes are active both in intact animals and in those undergoing the fission (*Cs-TIMP1*, *Cs-TIMP4-8*, *Cs-TIMP11*).

One of the key molecules of the mechanism of changing the MCT properties in echinoderms is tensilin. According to Keene, Trotter (unpubl., cited by Wilkie [39]), this protein plays an important role in increasing the stiffness of MCT. Previously, it was suggested that tensilin is similar in structure to TIMP [39]. Data of Clouse et al. [37] and our data agree with this assumption. It turned out that the transcripts of two *TIMP-like* genes of *C*. *schmeltzii* encode proteins, which are close to tensilins of other holothurians. In this connection, they were named *Cs-tensilin1* and *Cs-tensilin2*.

**Fig 4:**
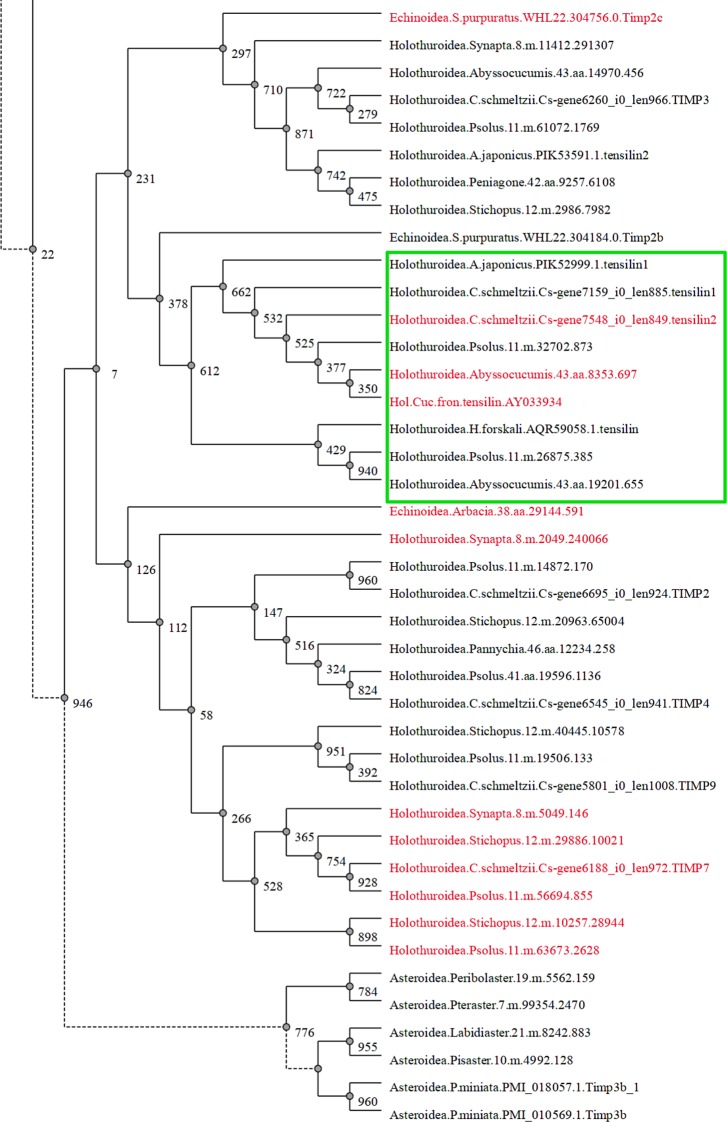
Part of phylogenetic tree constructed by maximum-likelihood method of TIMP proteins from 144 echinoderm and two non-echinoderm sequences (full version of the philogenetic tree is represented in S2 Fig). TIMP proteins with metzincin-binding interface are denoted by red color, green frame borders group of tensilins.

Currently, three proteins close to tensilin of *C*. *frondosa*—two for *A*. *japonicus* (tensilin1 and tensilin2) and one for *Holothuria forskali*—are found in the NCBI databases. Almost all the tensilins, including the tensilins of *C*. *schmeltzii*, are combined into a single group on the TIMPs tree (Fig 4). The only exception is tensilin2 of *A*. *japonicus*. An analysis showed that this protein has a low identity with tensilins, and, apparently, should not be termed “tensilin”. In addition, we analyzed those sequences that got into a group common with tensilins. All of them appeared to have the greatest similarity with tensilins of the holothurians *C*. *frondosa* and *H*. *forskali*. These are two proteins of *A*. *abissorum* (43.aa.8353.697 and 43.aa.19201.655) and two proteins of *Psolus sp*. (11.m.32702.873 and 11.m.26875.385). Hence, they also should be referred to as tensilins.

The functions of tensilins, as well as echinoderm TIMPs, can only be judged from their structure. Among nine tensilins, only three have the metzincin-binding interface. These are Cs-tensilin2, tensilin of *C*. *frondosa*, and one of proteins of *A*. *abissorum* (43.aa.8353.697) (Fig 4, S2 Fig). In this connection, it can be assumed that only these three proteins are able to bind with MMPs and inhibit their activity. However, *Cs-tensilin2* probably does not participate in the fission mechanisms, since the number of transcripts of this gene in the analyzed samples was below the threshold value accepted by us. At the same time, the *Cs-tensilin1* contigs are found in large quantities in both intact and dividing individuals (Table 2, S1 Table). For this reason, it can be assumed that *Cs-tensilin1* can be involved in regulation of asexual reproduction and, possibly, in the ECM transformation in holothurians.

All the detected tensilins belong to members of relatively young groups of holothurians [149] and, consequently, have formed within the class Holothuroidea. No similar proteins are found in Apodida (the most ancient order of holothurians), as well as in other echinoderms. The question that arises in this regard is as follows: how versatile are the mechanisms of MCT mutability in echinoderms? If tensilins participate in increasing the stiffness of MCT, they do this only in holothurians. In other echinoderms, this function must be performed by other molecules such as TIMPs.

Tensilins are found only in the species belonging to the order Dendrochirotida and a group of holothurians that were earlier combined into the order Aspidochirotida [149]. Dendrochirotids and aspidochirotids are distinguished by the presence of the quite thick body wall. The emergence of tensilins is supposedly associated with its formation. Indirectly, this assumption confirms the lack of tensilins in apodids [37], which have the body wall much thinner than that in dendrochirotids and aspidochirotids.

### Signaling pathways

As is known, the site of constriction formed in case of asexual reproduction in holothurians is a species-specific trait [16,150], and, obviously, should be marked by products of certain genes. In most animals, the anterior-posterior axis and regionalization of the body along the axis is determined by expression of genes of the Wnt and Hox families [151–154]. In this regard, a search for transcripts of *wnt* genes was performed in transcriptomes of *C*. *schmeltzii*. We found products of 10 out of the 12 *wnts* typical of holothurians [155]–*wntA*, *wnt1-7*, *wnt10*, *wnt16* (S1 Table). Transcripts of *wnt2* and *wnt4* were found only in dividing individuals, whereas *wnt1* and *wnt7* were revealed both in intact and in dividing holothurians (Table 3, S1 Table). The presence of products of these four genes at the site of fission may probably indicate the involvement of the Wnt signaling pathway in the regulation of asexual reproduction. In particular, they can determine the location of fission by forming a gradient of Wnt proteins along the anterior-posterior axis of animal. In addition, it is known that the Wnt signaling pathway plays an important role in regeneration in various animals [156–159]. Recently, it has been shown that some *wnt* genes are expressed during restoration of internal organs in holothurians [155,160,161]. In this regard, it can be assumed that this signaling pathway, along with the transcription factors (see below), is involved in regulation of fission and/or preparation of holothurian tissues for subsequent regeneration.

**Table 3:** Genes of components of signaling pathways expressing in intact and dividing individuals of *C*. *schmeltzii*.

Gene	intact	fission
*BMP2/4*	+	+
*BMP5/8*	+	+
*frizzled 1/2/7*	+	-
*frizzled 9/10*	-	+
*InhibinB*	+	+
*myostatin*	+	+
*smoothened*	-	+
*Sonic hedgehog*	+	+
*TGF-β 2*	+	+
*Wnt1*	+	-
*Wnt2*	-	+
*Wnt4*	-	+
*Wnt7*	+	-

Besides Wnt ligands, we found transcripts of genes encoding the receptors and messengers of this signaling pathway in transcriptome of. *C*. *schmeltzii*. In particular, there were transcripts of the genes *frizzled1/2/7*, *frizzled4*, and *frizzled9/10*. It is worth mentioning that products of *frizzled9/10* are found only in individuals that undergo fission and absent in intact animals (Table 3, S1 Table). This can mean that the *frizzled9/10* receptor and the Wnt signaling pathway, triggered through it, are involved in regulation of fission and/or preparation for the subsequent regeneration of internal organs.

Formation of ECM and its renewal are regulated by the TGF-β signaling pathway [162]. Transcripts of *TGF-β* were detected in both dividing and intact individuals of *C*. *schmeltzii* (Table 3, S1 Table). In addition to *Wnt* and *TGF-β*, products of genes of other signaling pathways–*BMP2/4*, *BMP5/8*, *myostatin*, *Shh*,–are found in *C*. *schmeltzii* (Table 3, S1 Table).

### Transcription factors

Products of a large number of transcription factors were found in the transcriptome of *C*. *schmeltzii*. This is not surprising, as many of them regulate physiological processes. In this regard, the factors that could be related to asexual reproduction and regeneration were analyzed. In holothurians, asexual reproduction occurs by the architomy type, and regeneration of the lost structures begins only after fission [3,16,20,21]. Nevertheless, products of the genes of 26 transcription factors that are related to regulation of morphogenesis are detected only in dividing *C*. *schmeltzii* (Table 4, S1 Table). Many of them, such as Tbx2/3, SoxD1, FoxK2, Runt, Krüppel-like and GATA factors, are activated during embryogenesis or regeneration in other animals. The presence of these factors in dividing holothurians indicates that preparation for the subsequent regeneration begins immediately during asexual reproduction. In addition, some genes involved in specification of endoderm and formation of the digestive system during development of animals are expressed only in dividing individuals. These are the genes *Sox9*, *SoxD1* [163,164], GATA4/5/6 [165] and a number of other ones (Table 4, S1 Table). Early expression of genes that regulate dedifferentiation and morphogenesis makes similar the mechanisms of architomy and paratomy, in which formation of the head and tail structures begins as early as in the process of fission [1,3,166].

**Table 4:** Transcription factors expressing in intact and dividing individuals of *C*. *schmeltzii*.

Gene	intact	fission	Gene	intact	fission	Gene	intact	fission
*AHR*	*-*	*+*	*HES1_1*	*+*	*-*	*Pax2/5/8*	+	-
*ALX1/3/4*	*+*	*-*	*HEY1/2/L*	*-*	*+*	*PINK1*	+	+
*ARID3*	*+*	*+*	*HIF1a*	*+*	*+*	*PKNOX*	-	+
*ARNT*	*-*	*+*	*HMGB2*	*+*	*+*	*PPARa*	+	-
*Ash1*	*+*	*+*	*HNF4*	*-*	*+*	*PRIKLE*	+	-
*Ash2*	*-*	*+*	*IRX4/6*	*+*	*-*	*PROX1/2*	-	+
*bHLH*	*+*	*-*	*JUN*	*+*	*+*	*PRX2*	-	+
*BHLHA15*	*+*	*+*	*KLF11*	*-*	*+*	*RARa*	+	+
*BIRC6*	*+*	*+*	*KLF3/8/12*	*+*	*-*	*RARb*	-	+
*CEBPa/b/d*	*-*	*+*	*KMT2A/B*	*+*	*-*	*REL*	+	-
*CEBPg*	*+*	*-*	*LBX*	*+*	*+*	*RFX1/2/3* (*RFX7*)	+	-
*Clock*	*-*	*+*	*LMO2*	*-*	*+*	*RUNT*	-	+
*CREB3l1/2*	*+*	*+*	*MAX*	*+*	*+*	*SALL1*	-	+
*CREB3l3/4*	*-*	*+*	*MBTD1*	*+*	*-*	*SCRT*	+	-
*CUX1*	*+*	*+*	*MLLT3*	+	+	*SFMBT2*	+	-
*DBP*	*+*	*+*	*MLXIP*	+	-	*SIX3*	+	-
*DGRX*	*+*	*-*	*MSX*	+	+	*SIX4*	+	+
*DMRT*	*+*	*-*	*MXI1*	+	+	*SMAD1/5/8*	+	-
*ELK1/3/4*	*-*	*+*	*MYF5*	+	-	*SOXD1*	-	+
*ERF*	*+*	*+*	*Nf-κB*	-	+	*SOX9*	-	+
*ESRRb*	*+*	*-*	*NKX2-1*	+	+	*SP2/4*	+	-
*EZH1/2*	*+*	*-*	*NPAS3*	+	+	*SP5*	+	-
*FOSL1*	*+*	*+*	*NR1H4*	+	+	*TBX2/3*	-	+
*FoxJ2/3*	*+*	*+*	*NR2E*	-	+	*TSC22D2*	+	-
*FoxK2*	*-*	*+*	*NR2F1/2*	+	+	*TULP4*	+	-
*FoxO*	*+*	*+*	*NSD1*	+	-	*ZEB1/2*	-	+
*GATA1/2/3*	*+*	*-*	*NSD2*	+	+	*ZFP410*	+	-
*GATA4/5/6*	*-*	*+*	*OSR*	-	+			
*GLIS1/3*	*+*	*+*	*p63*	+	+			

### Neuropeptides

Evidently, neuropeptides play an important role in regulation of properties of connective tissue in echinoderms [167]. In the holothurian *A*. *japonicus*, 20 bioactive peptides, causing changes in connective tissue properties and/or muscle contraction, were isolated from the body wall [57]. The transcripts encoding the precursors of the peptides–NGIWYamide and holokinin (homologue of bradykinin)–were found in *C*. *schmeltzii* (S1 Table). The NGIWYamide precursor is similar in structure with that of *A*. *japonicus* [57]. It incorporates the N-terminal signal peptide and five copies of the NGIWYG sequence that are located at the C-terminus of this protein. The NGIWYG sequences bounded by putative dibasic cleavage sites (KR). NGIWYamide in the holothurian *A*. *japonicus* shows the ability to increase stiffness of the body wall [167]. In *C*. *schmeltzii* the number of contigs in the samples was insignificant, thus indicating a low expression of this gene. This may mean that NGIWYamide does not participate in the ECM transformation during asexual reproduction.

Holokinin in *C*. *schmeltzii*, the same as in *A*. *japonicus* [57], is apparently a product of collagen destruction. The contig found in *C*. *schmeltzii*, coding the holokinin sequence, are blasted as collagen alpha-1(I) chain. This fragment encodes the C-terminus of collagen I/II/III containing the COLFI domain. Closer to the N-terminus of the contig, there is a sequence coding the Gly-X-Y motif, typical of collagens. The sequence encoding the holokinin PLGFLFR is located between it and the COLFI domain. Like that in *A*. *japonicus* [57], it is not bounded by putative monobasic or dibasic cleavage sites. Holokinin of *C*. *schmeltzii* is distinguished from that of *A*. *japonicus* by two amino acids in the middle part. According to Birenheide et al. [167], holokinins cause softening of the body wall in holothurians and, accordingly, can be involved in mechanisms changing the mechanical properties of MCT. When based on our data, it is difficult to judge whether holokinin is involved in the regulation of connective-tissue properties at fission. Contigs of *collagen I/II/III* occur in both intact and dividing individuals of *C*. *schmeltzii*. However, the collagen I/II/III itself is required for renewal of connective tissue, both in intact individuals and in asexual reproduction. Also, it is not clear whether the degradation of collagen with the release of holokinin occurs in this case. Nevertheless, this neuropeptide can be considered as a potential participant in the mechanism of changing the properties of connective tissue in holothurians.

## Conclusion

An analysis of the available literature and own data has shown that the composition of connective tissue in echinoderms is generally similar to that in vertebrates, but it has a number of significant differences. In echinoderms, no *tropoelastin*, *fibronectins*, and *tenascins* genes were found. In this connection, the structure and the mechanisms of renewal of ECM should have their own unique features that are yet to be clarified. Nevertheless, the main components of connective tissue, characteristic of vertebrates, are present in echinoderms. They have fibrillar collagens, collagens IX and XV/XVIII, fibrillins, fibulins, and thrombospondins. Molecules of these proteins and glycoproteins have a complex tertiary structure and can form both fibers and a three-dimensional network. Moreover, they have sites of binding with polysaccharides and other types of collagens, which allows them to form cross-links between fibrils. Obviously, the complexity of the ECM structure and the variety of intermolecular interactions predetermines also the complexity of the mechanisms of changing the connective tissue properties in echinoderms. Probably, these mechanisms depend not only on the number of cross-links, but also on the composition of ECM and the properties of constituent molecules.

Our study has shown that the fission process in holothurians is accompanied by significant physiological changes. The metabolism is increased, the nervous and immune systems are activated, and the structural changes occur in the fission zone. One of the significant indicators of morphological changes is the activation of the genes of laminins and nidogen, which probably an evidence of reorganization of epithelia. In the fission zone, the number of products of *collagen XV/XVIII* more as compared to that in intact individuals. This fact shows the importance of this type of collagen in the processes that take place during fission in holothurians, although the specific role of this protein is yet to be established. It is obvious that various proteases are involved in destruction of the body wall. In further studies of MCT transformation mechanisms, attention should be paid to such enzymes as ADAMTS, serine proteases, and cathepsin D. MMPs are apparently also involved in the modification and destruction of connective tissue in echinoderms; however, no qualitative change in their composition at fission was recorded during our study. To identify MMPs involved in ECM transformation, a more detailed study of their functions and dynamics of expression is necessary. The question of the participation of various neuropeptides in the transformation of connective tissue during asexual reproduction in echinoderms remains open. A small number of transcripts of *NGIWYamide precursor* in dividing individuals can be explained by the fact that this peptide is synthesized and accumulated at an earlier stage, when animal is still preparing for asexual reproduction.

It is obvious that various molecules that activate or inhibit proteases are also involved in the transformation of connective tissue. Holothurians have a wide range of these molecules that can regulate the activity of proteases at various levels. One of the well-known inhibitors is TIMPs. A total of 13 TIMP genes are found in *C*. *schmeltzii*, of which nine are expressed during fission. In this case, contigs of one of them, *Cs-TIMP10*, are detected only in dividing individuals. All the above facts confirm the data that TIMPs can participate in the transformation of ECM in echinoderms. Tensilins, being TIMP, apparently represent a separate group of genes, which have formed within the class Holothuroidea and which probably have a specific function, typical only of holothurians. The presence of tensilins can be associated with development of the thick connective-tissue body wall in these animals.

One of the distinguishing features of ECM in echinoderms, which is neglected by the existing hypotheses on MCT functions, is the presence of a large number of factors mediating the cell–cell and cell–matrix interactions. In this regard, it would be probably necessary to pay more attention to the structure and functions of fibrillins in echinoderms. It is possible that the fibrillin microfibril scaffold, like that of vertebrates, forms a niche for regulatory factors and mechanosensation. Conducting a signal from the extracellular microenvironment to competent cells can be a part of the mechanisms of MCT mutability.

In addition to the genes responsible for transformation of ECM, a large number of factors, which probably regulate the division of body into parts and the preparation of tissues for subsequent regeneration, are expressed in asexual reproduction. We could not identify the genes responsible for determination of the site of division. The most probable candidates for this role are genes of the Wnt familiy, but more studies are needed to confirm this assumption. The difference in the qualitative composition of the expressing transcription factors between intact and dividing holothurians will make it possible in the future to identify factors that regulate asexual reproduction. Moreover, the presence of transcripts of genes involved in regulation of morphogenesis of various tissues and organs may indicate that the preparation of tissues for the subsequent regeneration in holothurian begins immediately during fission.

## Supporting information

S1 Fig Length distribution of assembled contigs. (ZIP)

S2 Fig Phylogenetic tree constructed by maximum-likelihood method of TIMP proteins from 144 echinoderm and two non-echinoderm sequences. TIMP proteins with metzincin-binding interface are denoted by red color, green frame borders group of tensilins.
(ZIP)

S1 Table Best BLASTX hits obtained in similarity searches of contigs from *C*. *schmeltzii* versus the Swissprot and NCBI non-redundant protein databases. (ZIP)

S1 File Serine proteases of *C*. *schmeltzii* in MEGA6. (ZIP)

S2 File Matrix metalloproteinases of *C*. *schmeltzii* in MEGA6. (ZIP)

S3 File Aligned sequences of TIMPs of echinoderms in MEGA6. (ZIP)
