# Supplementary figures and images for: Molecular mechanisms of fission in echinoderms: Transcriptome analysis
Source: PLoS One. 2018 Apr 12;13(4):e0195836. doi: 10.1371/journal.pone.0195836 (PMC5897022; doi:10.1371/journal.pone.0195836)

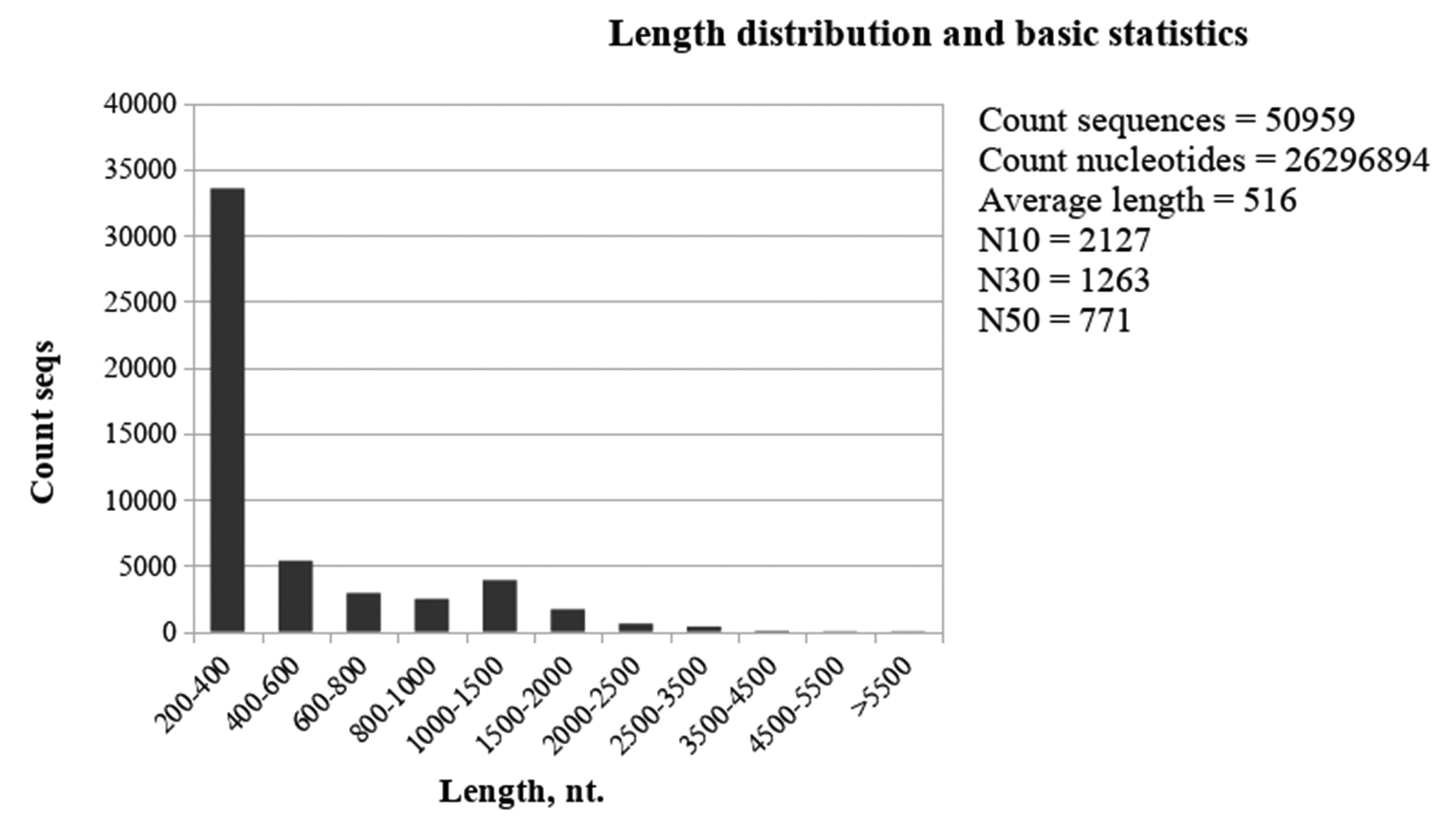

Supplement: S1 Fig — (ZIP) [file pone.0195836.s001.zip › Len_distr.tif]

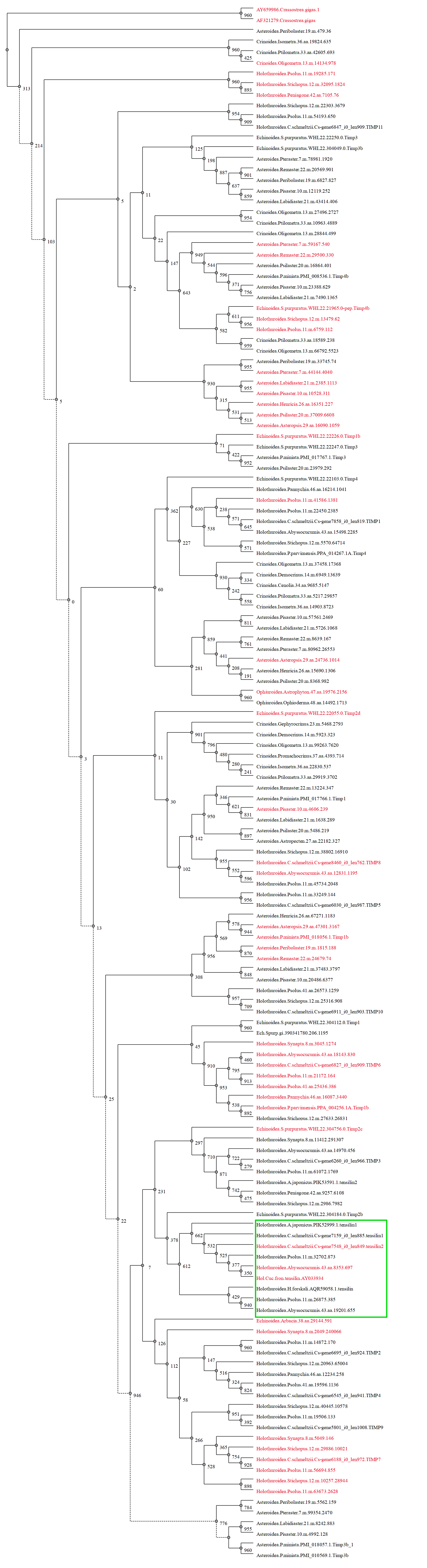

Supplement: S2 Fig — TIMP proteins with metzincin-binding interface are denoted by red color, green frame borders group of tensilins. (ZIP) [file pone.0195836.s002.zip › TIMP tree.png]
